# Determinants of use of health facility for childbirth in rural Hadiya zone, Southern Ethiopia

**DOI:** 10.1186/s12884-016-1151-1

**Published:** 2016-11-16

**Authors:** Netsanet Abera Asseffa, Fawole Bukola, Arowojolu Ayodele

**Affiliations:** 1 College of Health Sciences and Medicine, Wolaita Sodo University, Wolaita Sodo, Ethiopia; 2 Department of Obstetrics and Gynaecology, College of Medicine, Pan-African University, University of Ibadan, Ibadan, Nigeria

**Keywords:** Institutional delivery, Facility-based childbirth, Place of childbirth, Hadiya zone

## Abstract

**Background:**

Maternal mortality remains a major global public health concern despite many international efforts. Facility-based childbirth increases access to appropriate skilled attendance and emergency obstetric care services as the vast majority of obstetric complications occur during delivery. The purpose of the study was to determine the proportion of facility delivery and assess factors influencing utilization of health facility for childbirth.

**Methods:**

A cross-sectional study was conducted in two rural districts of Hadiya zone, southern Ethiopia. Participants who delivered within three years of the survey were selected by stratified random sampling. Trained interviewers administered a pre-tested semi-structured questionnaire. We employed bivariate analysis and logistic regression to identify determinants of facility-based delivery.

**Results:**

Data from 751 participants showed that 26.9% of deliveries were attended in health facilities. In bivariate analysis, maternal age, education, husband’s level of education, possession of radio, antenatal care, place of recent ANC attended, planned pregnancy, wealth quintile, parity, birth preparedness and complication readiness, being a model family and distance from the nearest health facility were associated with facility delivery. On multiple logistic regression, age, educational status, antenatal care, distance from the nearest health facility, wealth quintile, being a model family, planned pregnancy and place of recent ANC attended were the determinants of facility-based childbirth.

**Conclusion:**

Efforts to improve institutional deliveries in the region must strengthen initiatives that promote female education, opportunities for wealth creation, female empowerment and increased uptake of family planning among others. Service related barriers and cultural influences on the use of health facility for childbirth require further evaluation.

**Electronic supplementary material:**

The online version of this article (doi:10.1186/s12884-016-1151-1) contains supplementary material, which is available to authorized users.

## Background

Maternal mortality continues to be a major public health concern fifteen years after Millennium Development Goals (MDGs) were launched which hoped to reduce maternal mortality by 75% by 2015. However, Maternal Mortality Ratio (MMR) dropped by only 45% between 1990 and 2013 which is unsatisfactory progress in order to meet the goal [1] In 2013, sub-Saharan Africa had the highest MMR (510 per 100,000 live births) which was more than twice the global average [1].

Two thirds of maternal deaths occur around the time of childbirth; majority of these complications cannot be predicted by any high-risk approach since they occur at any time without any warning sign [2] Home delivery is believed to be a major factor contributing to such incidences. Hence, being in a health facility during childbirth is critical for saving the lives of mothers and their new-borns [1].

In low income countries, use of a health facility with a referral capacity is important in reduction of maternal mortality; however, in many developing countries deliveries occur outside the health facility. In sub-Saharan Africa, only 42.9% of deliveries occur in the health facility [3] When it comes to Ethiopia, utilization of health facility for childbirth is much lower, only 10% of childbirths occur in the health facilities; home deliveries are very common in the rural areas accounting for about 95% of all deliveries [4] Studies showed that various factors influence the utilization of health facility for childbirth; among them are socio-demographic factors, obstetric characteristics, socio-economic factors, and physical accessibility of the health facility [5–7].

The concept of model family has been employed in Ethiopia to promote adoption of facility based births among other health seeking practices [8] Model families are role models as well as change agents within the community. After adopting 12 of 16 government priority interventions a family will be recognized as a model family. The district health department is responsible for the recognition after the health extension worker acknowledges as the family has adopted at least 12 out of 16 government priority interventions. The 16 government priority interventions are vaccinating children, modern treatment to common childhood illnesses, using family planning, facility based delivery, sleeping under mosquito bed nets, building own latrine, antenatal care use, proper solid and liquid waste disposal, giving health education to others on harmful traditional practices, personal hygiene, healthy home environment, proper excreta disposal, food hygiene, control of insects and rodents, nutrition, and adolescent reproductive health [8].

Analysis of Ethiopian Demographic and Health Survey 2011 showed that the proportion of women who had delivered in the health facility was five times higher among urban residents as compared to those in rural areas [9] In order to address this variability among urban-rural dwellers and extremely low utilization of health facility for childbirth, it is important to understand the determinants. In addition, despite the fact that there has been an increase in the number of health facilities and providers in the rural areas of Ethiopia, women continue to deliver outside the health facility [8].

Hence, this study was designed to assess the proportion of facility based deliveries and determine factors influencing the utilization of health facility for childbirth in rural Hadiya zone, southern Ethiopia.

## Methods

A community based cross-sectional study was conducted in two rural districts of Hadiya zone, southern Ethiopia. In the zone, there were 280 Health Posts (HPs), 60 rural Health Centres (HCs) and one hospital serving a population of nearly 1.2 million. Hadiya zone is divided into 11 districts for administrative purposes. The vast majority of the population are Hadiya in ethnic group and they earn their living through rain fed agriculture.

The Lemo district has a population of 144, 244 with about 33,176 childbearing women whereas the population of Gombora district is 113, 004 with about 26,330 childbearing women. Lemo district has 7 HCs and 33 HPs while Gombora has 6 HCs and 23 HPs. In both districts each rural *kebele* (the lowest administrative unit in Ethiopia), has one HP. About 98% of Lemo district and 100% of Gombora district populations are rural dwellers [10, 11].

The study population comprised women who reside in the *kebeles* selected from the two rural districts (Lemo and Gombora) and had delivered in the last three years preceding the study. The sample size was calculated using single population proportion determination formula; by taking point estimates of institutional childbirth of southern region which was 6.2 [4] Other inputs considered for the sample size determination were: 95% confidence level, design effect (deff) of 2, precision of 2.5% and 5% non-response rate which makes a total of 756 respondents.

Stratified random sampling was used to select the study units. Firstly the study areas were divided into two groups; six of which were far (>20 km) from and four that were close (within 5–20 km) to the zonal capital. In order to have a good representation, one district was selected from each group using lottery method.

Then all *kebeles* in the selected districts were grouped into five based on geographical direction, afterwards one *kebele* was selected from each group. Then enumeration was done in the selected *kebeles by going* house to house in order to identify eligible mothers (women who had given birth in the last three years preceding the survey; regardless of the current pregnancy status or outcome of the previous pregnancy or place of delivery) and 2,474 women were found to be eligible and all houses were coded. Thereafter, 756 women were randomly selected for the interview using proportional allocation. Regarding weighting a simple balancing was done and each observation had a weight of 1 as all eligible mothers were identified by enumeration and sampling procedure was done carefully.

A structured questionnaire was adopted after reviewing relevant studies done previously Additional file 1). The 50-item questionnaires had 5 sections. It was constructed in English language and then translated into Hadiyigna language. Before actual collection of data, a written informed consent was obtained from all respondents after explanation of the purpose, objective, risk and benefit of the study.

Also a pre-test was done in similar district in 10% of the respondents which was 75 and necessary corrections were made. The interviews were conducted in a convenient, quiet and private place for the mother. It took 25–40 min to complete the questionnaire. Data were collected by ten trained female data collectors who were High School Teachers and had Bachelor degrees. The questionnaires were checked for consistency and completeness before being entered into EPI data version 3.1 software for cleaning and exploration, and analysed using SPSS version 20.0.

The responses concerning the outcome variable, place of delivery were three: health facility, home and on their way to the health facility. We had only two (0.0027%) of respondents who gave birth on their way to the health facility, we found these to be very small to be analysed alone therefore we added them to the home deliveries. Finally, home births were coded zero and health facility deliveries were coded one. We used principal component analysis method to generate wealth quintiles. It was based on household assets, dwelling characteristics, any livestock, agricultural land and others. Planned (a pregnancy which the woman becomes pregnant after intending to be pregnant or a pregnancy which a woman and her partner had discussed and decided in advance) and unplanned pregnancies (a pregnancy which the woman becomes pregnant without intending to or a pregnancy a woman and her partner had not discussed and decided in advance) were among the factors.

Summarization was done using percentage, tables, figures and summary statistics. Binary logistic regressions were used to calculate Crude Odds Ratios (COR) for birth in a health facility and 95% Confidence Intervals (CI). Variables which were statistically significant at *p* < 0.05 on bivariate analysis were entered into multiple logistic regression model. Variables having strong correlation were planned to be excluded from the final model. Parity and place ANC attended were not included in the final model as it had strong correlation with age and numbers of ANC attendances respectively. Odds ratio was adjusted for all other variables. The significance level was set at p-value less than 0.05. The Hosmer-Lemshow goodness-of-fit test was also checked. Ethical approval letter was obtained from South Regional Health Bureau Ethics Committee.

## Results

Of the total respondents, 391 (52.1%) were from Lemo district while 360 (47.9%) were from Gombora district. The mean age of the respondents was 28.64.6 SD. Three hundred and nineteen (42.5%) women were in the age range of 25–29 years, 729 (97.1%) were in union, 696 (92.7%) were protestant religion followers, 660 (87.9%) were Hadiya ethnic group, 465 (61.9%) respondents and 404 (53.8%) husbands had primary education. Concerning the wealth status, 150 (20.0%) were in the poorest quintile while 145 (19.3%) were in the richest quintile (Table 1).

**Table 1:** Socio-demographic characteristics of respondents in rural Hadiya zone, southern Ethiopia

Variables	*n* (%)
District	
Lemo	391 (52.1)
Gombora	360 (47.9)
Age in years	
< 25	123 (16.4)
25–29	319 (42.5)
30–34	212 (28.2)
> 34	97 (12.9)
Marital status	
Not in Union	22 (2.9)
In Union	729 (97.1)
Religion	
Protestant	696 (92.7)
Orthodox	10 (1.3)
Muslim	35 (4.7)
Others^a^	10 (1.3)
Ethnicty	
Hadiya	660 (87.9)
Silte	35 (4.7)
Amhara	16 (2.1)
Gurage	19 (2.5)
Others^b^	21 (2.8)
Educational status	
No education	167 (22.2)
Primary	465 (61.9)
Secondary	78 (10.4)
Above secondary	41 (5.5)
Husbands’ educational status	
No education	77 (10.6)
Primary	404 (55.4)
Secondary	171 (23.5)
Above secondary	77 (10.6)
Wealth quintile	
Poorest	150 (20.0)
Poor	143 (19.0)
Middle	158 (21.0)
Rich	155 (20.6)
Richest	145 (19.3)

^a^Catholic (6), Hawariyat (4). ^b^ Kembata (13), Beteisrael (7), Halaba (1). Preacher (8), Cart driver (3). Family member (4), TBA (1)

A total of 677 (90.1%) visited ANC among whom 357 (52.7%) attended less than four times, 355 (52.4%) mothers attended ANC at health post where the service was mostly provided by health extension workers. During their ANC visit, 88 (13.0%) of them were told that they had problem/s related to pregnancy. Out of the total childbirths, 379 (50.5%) were unplanned pregnancies. Eight seven (11.6%) of respondents had parity one while 317 (42.2%) were parity five and above.

Five hundred eighty (77.2%) had some form of birth preparedness and complication readiness meaning that they planned for normal birth as well anticipated actions needed in case of emergencies; most (75.1%) mentioned that they prepared clean items for childbirth and post-partum period. Six hundred fifty six (87.4%) had no history of pregnancy related complications (Table 2).

**Table 2:** Obstetric characteristics of respondents in rural Hadiya zone, southern Ethiopia

Obstetric characteristics	*n* (%)
ANC visit during most recent pregnancy	
Yes	677 (90.1)
No	74 (9.9)
Number of ANC visit/s (*n* = 677)	
1–3	357 (52.7 %)
4 or above	320 (47.3)
Place of most recent ANC attendance	
Health Post	355 (52.4)
Health Centre	314 (46.4)
Hospital	7 (1.0)
Private clinic	1 (0.1)
Last pregnancy	
Planned	372 (49.5)
Unplanned	379 (50.5)
Problem/s identified during ANC visit	
Yes	88 (13.0)
No	589 (87.0)
History of pregnancy related complication/s	
Yes	95 (12.6)
No	656 (87.4)
Birth preparedness and complication readiness	
Yes	580 (77.2)
No	171 (22.8)
Parity	
1	87 (11.6)
2–4	347 (46.2)
> 4	317 (42.2)

During their most recent childbirth, 549 (73.1%) delivered at home while 202 (26.9%) at health facility. Out of the total respondents, 281 (37.4%), 221 (29.4%), 35 (4.7%), 7 (0.9%) and 1 (0.1%) were assisted by TBA, family members, friends, Health Extension Workers (HEW) and mother herself respectively while 206 (27.5%) were attended by skilled providers.

Various reasons were mentioned for choosing home delivery: 280 (50.6%) women mentioned that pervious childbirths were successful without attending a health facility. Two hundred forty five (44.3%) mentioned that pregnancy was normal. One hundred thirty nine (25.1%) stated that labour was urgent. Also, thirty seven (6.7%) mentioned lack of money. Twenty four (4.4%) mentioned faith in God that nothing would happen. Eighteen (3.3%) mentioned that health facility was too far. Twenty six (4.7%) stated no transportation. Eight (1.4%) mentioned labour was at night. Nineteen (3.4%) mentioned other reasons.

Out of the total respondents, 626 (83.4%) were visited by health extension workers at their homes. Concerning being a model family, 605 (80.6%) had never been recognized as a model family whereas 146 (19.4%) had been recognized.

Regarding physical accessibility, the average distance from a household to the nearest health centre was 3.0 km1.4 SD and 18.0 km11.1 SD to the hospital. Concerning the available (usual) transportation to take to the health facility, 745 (99.2%) mentioned foot. Among mothers who had the last childbirth at health facility, 74 (36.6%) used ambulance to go to the health facility, 47 (23.3%) went by cart, 25 (12.4%) by locally made stretcher, 24 (11.9%) used tri-cycle/Bajaj, fourteen (6.9%) went on foot, thirteen (6.4%) used public transportation and five (2.5%) used other transportation mechanism to reach the health facility.

## Determinants of use of health facility for childbirth

In the bivariate analysis, most explanatory variables were found to be associated with the use of health facility for childbirth at *p* < 0.05. Factors associated with utilization of health facility for childbirth included age, women’s level of education, husband’s level of education, possession of radio, antenatal care, place of recent ANC attended and planned pregnancy. Other factors such as wealth quintile, parity, birth preparedness and complication readiness, being a model family and distance from the nearest health facility were also among the associated factors (Table 3). In our study, variables such as marital status, religion, ethnicity, occupation, having history of pregnancy related complications and having problems related to pregnancy were not statistically significant.

**Table 3:** Bivariate and multiple logistic regression of determinants of use of health facility for childbirth

Variables	Place of child birth		COR (95 % C.I)	AOR (95 % C.I)	*P*-value
	HF *n* (%)	Home *n* (%)			
Age (years)					
< 25	50 (40.7)	73 (59.3)	3.4 [1.9–4.8]*	1.9 [1.1–3.6]	0.035
25–29	95 (29.8)	224 (70.2)	1.9 [1.3–2.7]*	1.3 [0.8–2.0]	0.333
30 and above	56 (18.4)	252 (81.6)	ref		
Educational status					
No education and primary	125 (19.8)	507 (80.2)	ref		
Secondary and above	77 (64.7)	42 (35.3)	7.4 [4.9–11.4]*	2.3 [1.3–3.9]	0.004
Husband’s educational status					
No education and primary	93 (19.3)	388 (80.7)	ref		
Secondary and above	105 (42.3)	143 (57.7)	3.0 [2.2–4.3]*	1.3 [0.8–2.0]	0.274
Possession of radio					
Yes	140 (39.3)	216 (60.7)	3.5 [2.5–4.9]*	1.3 [0.8–2.1]	0.315
No	62 (15.7)	333 (84.3)	ref		
4 or more ANC visits					
Yes	146 (45.6)	174 (54.4)	4.9 [3.4–7.1]*	2.7 [1.7–4.1]	<0.001
No	52 (14.6)	305 (85.4)	ref		
Place of recent ANC attended					
Health post	65 (18.3)	290 (81.7)	ref		
HC and hospital	133 (41.3)	189 (58.7)	3.1 [2.2–4.4]*	1.8 [1.2–2.7]	0.009
Last pregnancy planned					
Yes	152 (40.9)	220 (59.1)	4.5 [3.2–6.5]*	2.4 [1.5–3.8]	<0.001
No	50 (13.2)	329 (86.8)	ref		
Birth preparedness and complication readiness					
Yes	190 (32.8)	390 (67.2)	6.4 [3.5–11.9]*	1.9 [0.9–4.1]	0.091
No	12 (7.0)	159 (93.0)	ref		
Model family					
Yes	60 (41.1)	86 (58.9)	2.3 [1.6–3.3]*	1.7 [1.1–2.7]	0.043
No	142 (23.5)	463 (76.5)	ref		
Distance from the nearest HF					
< 2kms	60 (45.1)	73 (54.9)	5.9 [2.6–13.4]*	3.1 [1.2–7.9]	0.022
2–4kms	134 (24.3)	418 (75.7)	2.3 [1.1–5.0]*	1.7 [0.7–4.2]	0.23
> 4kms	8 (12.1)	58 (87.9)	ref		
Wealth quintile					
Poorest	8 (5.3)	142 (94.7)	ref		
Poor	18 (12.6)	125 (87.4)	2.6 [1.1–6.1]*	1.6 [0.6–4.1]	0.312
Middle	35 (21.2)	123 (78.8)	5.0 [2.2–11.3]*	1.9 [0.8–4.7]	0.158
Rich	56 (36.1)	99 (63.9)	10.0 [4.6–22.0]*	3.4 [1.4–8.4]	0.008
Richest	85 (58.6)	60 (41.4)	25.1 [11.5–55.1]*	4.3 [1.7–11.1]	0.003

The *p*-values of multivariable analysis were based on Likelihood ratio test

Parity was not included in the final model because of its strong correlation with Age, Odds ratio was adjusted for all other variables in the table, The Hosmer-Lemshow goodness-of-fit test was checked

*COR* Crude Odds Ratio, *AOR* Adusted Odds Ratio

*significant at *p*-value less than 0.05 on bivariate logistic regression

On the multiple logistic regression, age, educational status, 4 or more antenatal clinic attendance, distance from the nearest health facility, wealth quintile, being a model family, planned pregnancy and place of recent ANC attended remained significant (Table 3).

Mothers whose ages were less than 25 years had about two times the odds of use of health facility for childbirth (AOR = 1.9, 95% CI [1.1–3.6]). Likewise, mothers with secondary or higher levels of education were more likely to give birth in the health facility (OR = 2.3, 95% CI [1.3–3.9]).

Regarding the obstetric characteristics of the women; women who attended ANC four times or more had approximately three times the odds of delivery in the health facility as compared to women who had childbirth at home (AOR = 2.7, 95% CI [1.7–4.2]). Type of pregnancy (planned versus unplanned) was also found to be an independent predictor of place of childbirth; mothers who had planned their recent pregnancy were more than two times more likely to deliver in the health facility than their counterpart (AOR = 2.4, 95% CI [1.5–3.8]). Women were asked where they attended the most recent ANC; those who attended the health centre or hospital had about two times the odds of childbirth in the health facility than those who attended the health post.

Factors related to economic status also remained significant on multiple logistic regression. Facility delivery was more likely among mothers who were in the wealthier quintiles than those in the poorer quintiles. The odds of delivering in the health facility for wealthiest women were more than four times likely than that of the poorest women (AOR = 4.3, 95% CI [1.7–11.1]).

**Fig. 1:**
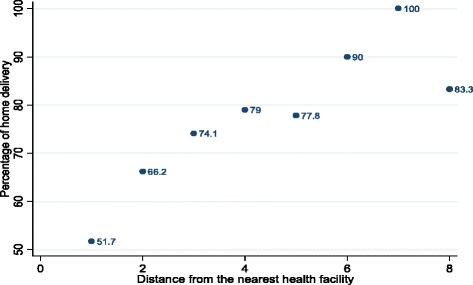
Distance from the nearest health facility and percentage of home births among rural women who delivered in the last three years preceding the survey, rural Hadiya zone, southern Ethiopia

A family that had been recognized as model was found to be about two times more likely to have childbirth at health facility than one that had not been recognized (AOR = 1.7, 95% CI[1.1–2.7]). Respondents who were within less than two kilometres of the nearest health facility had three times the odds of delivering in the health facility than those residing more than four kilometres away from the nearest health facility (AOR = 3.3 95% CI [1.2–8.7]). The implication of this finding is that the proportion of home delivery increases as the distance from the nearest health facility increases (Fig. 1).

## Discussion

A total of 751 women were interviewed in two rural districts of Hadiya zone, southern Ethiopia. Out of the total respondents, 43.7% attended ANC four or more times. Half (50.6%) of all deliveries were unplanned pregnancies. During their most recent delivery, 73.1% of the respondents delivered at home while 26.9% delivered in a health facility. Most (37.4%) of all deliveries were attended by traditional birth attendants. Among the reasons for home delivery, 280 (50.6%) mentioned their previous successful childbirth without attending health facility.

Analysis of this study showed that facility delivery rate was 26.9%. Although the figure is low it is considerably higher than other studies done in Ethiopia [4, 6, 12–15] One possible explanation can be that the Ethiopian government has implemented high impact interventions in the rural areas which has resulted an increase in institutional childbirth by 6.6% per annum [16] Also as the country is on the last lap of MDG5 target achievement, many governmental and non-governmental organizations are working towards improvement of maternal health.

On the contrary, utilization of health facility for childbirth is much lower than studies done in Rwanda where 89.8% delivered in a the health facility [17] Likewise, 47% in Kenya, 45.4% in Uganda, 60% in Nigeria and 74.5% in Tanzania had their last childbirth in a health facility [17–21] This variability could be explained by differences in levels of education, wealth distribution, awareness of risks and benefits of facility delivery. Also the studies may have focused on urban settings.

Our study revealed that the younger the mother’s age the more likely to utilize health facility for childbirth. In the multiple logistic regression, those who were aged less than 25 were about two times more likely to deliver in the health facility as compared to those aged 35 years and above (AOR = 1.9, 95% C.I [1.1–3.6]). This finding is inconsistent with other reports in which the older mothers were more likely to deliver in the health facility [12, 22, 23] This discrepancy might appear because of the fact that the older women in our study were less educated and might respect the norms whereby home delivery is customary; previous successful deliveries may also enhance their confidence. On the other hand, the finding is similar to the findings of a study from Bahirdar, Northwest Ethiopia [13].

In our study, variables such as marital status, religion and ethnicity had no significance. This might be because of the homogeneity of the respondents; 97.1% were married, 92.7% were protestant religion followers and 87.9% were Hadiya in their ethnic group.

This study has confirmed previous reports that maternal education is one of the most strongly and consistently associated factors with the facility childbirth [12, 15, 18, 22, 24, 25] Likewise, in our study women who had secondary education and above were twice likely to seek health facility delivery than those with no or primary education (AOR = 2.3, 95% C.I [1.3–3.9]). This might be because educated women tend to be aware of the benefits of maternal health services, likely to know risks of home delivery and able to afford costs necessary. Concerning the significance of husband’s educational status, no association was found between husband’s education level and utilization of health facility for childbirth (AOR = 1.3, 95% C.I [0.8–2.0]).

The proportion of women who had at least one ANC visit was impressive, 90.1% of them reported attendance. This figure is much higher than the regional rate of 40.8% and the national rate of 42.5% for any ANC visit [4] The reason for this significant variation could be physical accessibility of Health Posts where more than half of the women attended at least one ANC. The coverage of Health Post is 100% in both districts of our study [10, 11] However, only 47.3% of them reported 4 or more ANC visits. The reason could be one of the measurements of quality of ANC services is the qualification of the provider.

In our study 52.3% of women were attended by Health Extension Workers whose training program is of low quality, short duration and heterogeneous and their role in the maternal health remains controversial. The other possible explanation can be that the rural Hadiya zone women tend to feel confident and show less desire towards attending ANC once she gets the first dose of tetanus toxoid injection and iron folate.

On multiple logistic regression, women who had 4 or more ANC visits were approximately three times more likely to give birth in the health facility (AOR = 2.7, 95% C.I [1.7–4.1]). It is consistent with the findings of previous studies [12, 17, 23, 25–27] The reason for this consistency can be due to the fact that frequent visits to the health facility fostered better interaction with the health worker thus enhancing greater opportunities to recommend facility delivery, check health status of women and enabling the women to become more accustomed to the health system.

Our finding revealed that place of the recent ANC attendance was an independent predictor of place of childbirth. Women who attended the recent ANC at health facilities other than Health Post had about twice the odds of giving birth in the health facility (AOR = 1.8, 95% C.I [1.2–2.7]). This shows that those who were attended by skilled providers were more likely to have childbirth in the health facility as compared to those who attended Health Posts where unskilled providers (HEWs) are stationed. The reason could be that skilled providers provide high quality ANC service compared to less skilled counterparts.

Having planned versus unplanned pregnancy and its impact on place of childbirth was assessed in our study. We found that those who planned the pregnancy were more than two fold more likely to use health facility for childbirth than those unplanned pregnancies (AOR = 2.4, 95% C.I [1.5–3.5]). To the best of our knowledge, this finding does not seem to have been previously reported in the literature. Nevertheless, we concluded that women with unintended pregnancy are less likely to seek professional care as compared to those who are eager to have a child.

Among those who were para one, 42.5% of them used a health facility for childbirth, compared to 14.4% of women who delivered their fifth child in a health facility. This finding corroborates the findings of previous studies [7, 18, 22, 23, 25, 28, 29] This consistency could be due to the previous experiences women had which made them feel more confident and to assume that attending health facility is not necessary. It is worth noting that in our study most (51%) respondents who delivered at home mentioned successful previous birth as reason for not utilizing health facility for childbirth.

No significant relationship was found between possession of radio and or TV and use of health facility for childbirth (AOR = 1.3, 95% C.I [0.8–2.1]). This finding is inconsistent with findings from Pakistan [22] where about 58% of women who had frequent exposure to mass media attended health facility for childbirth. It also disagrees with study done in a Holeta, central Ethiopia [7], but it is in line with a study done in western Ethiopia [30] The variability might be caused by the fact that most media in the region as well in the nation broadcast via Amharic (National language of Ethiopia) which most rural Hadiya zone women do not speak.

The other independent factor which was strongly associated with the use of health facility for childbirth was wealth quintile. Those in the poorest quintile were four times less likely to deliver in the health facility as compared to women in the richest quintile (AOR = 4.3, 95% C.I [1.7–11.1]). This is in line with other studies done previously [5, 18, 21–24] Although maternity care is free at all levels of health facility in Ethiopia [4], socioeconomic condition remains a determinant factor. This might be due to the fact that wealthier women tend to afford costs necessary for transportation and other informal provider fees. In our study, we considered distance to the nearest health facility and found that women who resided within less than 2 km were three times more likely to deliver in the health facility as compared to those residing more than 4 km from the nearest health facility (AOR = 3.1, 95% CI [1.2–7.9]).

This study revealed that being a model family as an independent predictor of place of childbirth. Women who had ever been recognized as a model family had about two times the odds of giving birth in the health facility than those that had never been recognized (AOR = 1.7, 95% CI [1.1–2.7]). This might be because model families are expected to be role models in many aspects to the villagers as they are most celebrated in rural areas of Ethiopia.

One possible limitation of this study may be error in the distance measurement due to lack of Global Positioning System (GPS). However, the sample included in the analysis is satisfactory and we believed that all possible factors that influence facility delivery were captured.

## Conclusion

In order to address low coverage of facility delivery behavioural change communication would be paramount so as to improve women’s awareness about importance of delivery care and possible complications of childbirth. Good quality of antenatal care is also vital. The study revealed that an unplanned pregnancy was an independent predictor, therefore family planning programs should target those exposed to unwanted pregnancy.

Efforts to improve institutional deliveries in the region must strengthen initiatives that promote education, opportunities for wealth creation, female empowerment and increased uptake of family planning among others. It would be good to further study service related barriers and cultural influences on the use of health facility for childbirth.

## Additional file

Additional file 1: Questionnaire. (DOCX 56 kb)

## Abbreviations

ANC: Antenatal care
AOR: Adjusted odds ratio
CI: Confidence interval
HC: Health centre
HEP: Health extension program
HEW: Health extension worker
HP: Health post
MDG: Millennium development goal
MMR: Maternal mortality ratio
OR: Odds ratio
SD: Standard deviation
SPSS: Statistical package for social sciences
TBA: Traditional birth attendant
