# Supplementary material for: Determinants of use of health facility for childbirth in rural Hadiya zone, Southern Ethiopia
Source: BMC Pregnancy Childbirth. 2016 Nov 16;16:355. doi: 10.1186/s12884-016-1151-1 (PMC5112737; doi:10.1186/s12884-016-1151-1)
Supplement: Additional file 1: — Questionnaire. (DOCX 56 kb) [file 12884_2016_1151_MOESM1_ESM.docx]

Additional file 1

| Section I: - Respondent’s Socio-demographic Characteristics | | | | | | | |  |
| --- | --- | --- | --- | --- | --- | --- | --- | --- |
| No | | | Question | | Response | | Skip |  |
|  | | | Place of residence  District | | Lemo……………………1  Gombora………………….2 | |  |  |
|  | | | How old are you? | | _________ (in years) ......................1 | |  |  |
|  | | | What is your current marital status? | | Single………………………………………1  Married……………………………..……...2  Divorced…………………….……………..3  Widowed……………..…………………….4 | |  |  |
|  | | | What is your religion? | | Protestant ……………………...…………...1  Orthodox………………….………………...2  Muslim…,,,,………………………………....3  Other Specify________________ ...…….....4 | |  |  |
|  | | | To which ethnic group do you belong? | | Hadiya………………………………………1  Kembata………………………………….….2  Amhara…………………….………..….……3  Other Specify_________________................4 | |  |  |
|  | | | What is the highest level of school you attended? | | No education……………………………….1  Primary……………………………………..2  Secondary…………………………………..3  Above Secondary…………………………..4 | | 8 |  |
|  | | | What is the highest grade you completed at that level? | | ________________________________.........1 | |  |  |
|  | | | What is the highest level of school your husband attended? | | No education……………………………….1  Primary……………………………………..2  Secondary………………………..…………3  Above Secondary…………………………..4 | |  |  |
|  | | | What kind of work do you mainly do? (record only one response) | | No work …………………………………1  Farming ………………………………….2  Trading/Selling ………………………….3  Craftsmanship …………………………..4  Government employee………………….5  Student ………………………………….6  Labourer/Casual worker ……………….7  Other specify ___________________...8 | |  |  |
|  | | | What kind of work does your husband mainly do? (record only one response) | | No work ………………………………1  Farming ……………………………….2  Trading/Selling ………………………..3  Craftsmanship …………………………4  Government employee…………………5  Student ………………………………….6  Labourer/Casual worker…………………7  Other specify ___________________.....8 | |  |  |
|  | | | If you are needed to go to health facility for childbirth, do you first have to ask someone’s permission? | | Yes…………………………………………1  No………………………………………….2 | |  |  |
|  | | | For your last childbirth, who decided where you give childbirth? | | Woman……………………………1  Husband ……………………………………2  Jointly……………………………………….3  Mother in law……………………………….4  Health extension worker…………………….5  Others specify_________________ | |  |  |
|  | | | What is the main source of drinking water for your family?  *Note:-If more than one record the most usual one only* | | Piped water  Piped to dwelling………………………1  Piped to yard/plot….…………………..2  Public Tap/stand Pipe………………….3  Bore-hole...…………………………………4  Dug well  Protected dug well…………………5  Unprotected dug well……...………6  Spring  Protected Spring …………………...7  Unprotected Spring ……...…………8  River or Stream……………………………..9  Other specify______________________...10 | |  |  |
|  | | | Main material of the dwelling floor  *RECORD OBSERVATION* | | Natural floor  Earth/Sand………………………………1  Dung…………………………………….2  Rudimentary floor  Wood planks……………………………3  Palm/Bamboo  Finished floor  Asphalt strip…………………………….4  Ceramic tiles…………………………….5  Cement…………………………………..6  Carpet……………………………………7  Other specify________________________..8 | |  |  |
|  | | | Main material of the dwelling roof  *RECORD OBSERVATION* | | Natural roofing  No roof . . . . . . . . . . . . . . . . . . . . . . . .1  Thatch/leaf/mud . . . . . . . . . . . . . . . . .2  Rudimentary roofing  Rustic mat/plastic sheets . . . . . . …….3  Wood planks . . . . . . . . . . . . . . . . . . . . .4  Finished roofing  Corrugated iron /metal . . . . . . . . . . . . . 5  Wood . . . . . . . . . . . . . . . . . . . . . . . . . . 6  Asbestos/cement fiber….. ....... . ……..7  Cement/concrete……. . . . . . . . . . . . . . 8  Other specify………………………………9. | |  |  |
|  | | | How many rooms in your household are used for sleeping? | | ______________________________............1 | |  |  |
|  | | | Does your household have? (Tick all appropriate). | | Electricity…………………………………..1  A watch/clock………………………………2  A radio………………………………………3  A television………………………………….4  A mobile telephone………………………….5  A non- mobile telephone……………………6  A refrigerator………………………………..7  A table……………………………………….8  A bed with cotton/sponge/spring mattress….9  A kerosene lamp/pressure lamp…………...10 | |  |  |
|  | | | Does any member of household own: | | Bicycle………………………………………1  Motorcycle/scooter...………………………..2  Animal drawn cart…………………………...3 | |  |  |
|  | | | What kind of toilet facility do members of your household usually use?  Record Observation | | Pit latrine  Ventilated improved pit latrine (vip). . . . 1  Pit latrine with slab . . . . . . . . . . . . . . . . 2  Pit latrine without slab/ open pit . . . . . . 3  No facility/bush/field………………………4  Other specify____________________.........5 | |  |  |
|  | | | What type of fuel does your household mainly use for cooking? | | Kerosene…………………………………….1  Charcoal …………………………………….2  Wood ………………………………………..3  Animal dug…………………………………4  No food cooked in household …………….5  Other specify ____________________......6 | |  |  |
|  | | | Does any member of this household own any agricultural land? | | Yes . . . . . . . . . . . . . . . . . . . . . . . . . . . . . . . . . 1  No . . . . . . . . . . . . . . . . . . . . . . . . . . . . . . . . . 2 | | 23 |  |
|  | | | How many hectares of agricultural land do members of this household own? | | ______________hectares. | |  |  |
|  | | | Does this household own any livestock, herds, other farm animals, or poultry? | | Yes . . . . . . . . . . . . . . . . . . . . . . . . . . . . . .1  No. . . . . . . . . . . . . . . . . . . . . . . . . . . . . . ... ..2 | | 25 |  |
|  | | | How many of the following animals do this household own?  IF NONE, ENTER '00'.  IF MORE THAN 95, ENTER '95'.  IF UNKNOWN, ENTER '98 | | Cows/Bulls/Oxen . . . . …  Horses/Donkeys/Mules . . . . ……  Goats . . . . . . . . . . . . . .  Sheep . . . . . . . . . . . . . . . . . . . . .  Chickens . . . . . . . . . . .  Beehives…………………………… | |  |  |
| Section II: - Respondent’s Antenatal Care Utilization | | | | | | | |  |
|  | | | Did you attend ANC during your recent pregnancy? | | Yes…………………………………………1  No……………………………..…………...2 | | 31 |  |
|  | | | How many ANC visit/s have you had? | | ________________________________.....1 | |  |  |
|  | | | Where did you attend your recent ANC? | | Health Post…………………………………1  Health Centre………………………………2  Hospital…………………………………….3  Private clinic………………………………..4  Other Specify……………………………….5 | |  |  |
|  | | | Who gave you the ANC service? | | Health extension worker……………………1  Nurse………………………………………..2  Midwife……………………………………..3  Doctor……………………………………….4  Other specify………………………………..5 | |  |  |
|  | | | During your ANC visit/s were you told as you have had any problem related to pregnancy? | | Yes…………………………………………1  No………………………………………….2 | | 31 |  |
|  | | | What was that problem you were told? | | High blood pressure………………………..1  Multiple pregnancy…..……………………..2  Anaemia……………….…………………….3  Swelling of leg……………………………….4  Other specify_____________________........5 | |  |  |
|  | | | When you got pregnant with your last baby, have you had a plan to get pregnant? | | Yes ………………………………………….1  No…………………………………………...2 | |  |  |
| Section III:- Respondent’s Recent Childbirth service utilization | | | | | | | | |
|  | | | How many childbirth/s have you had? | | __________________________..................1 | |  | |
|  | | | Have you made any kinds of Birth preparedness and complication readiness? | | Yes ………………………………………….1  No…………………………………………...2 | | 35 | |
|  | | | What kinds of preparations were you made for your recent childbirth? (Tick all appropriate) | | Money were saved…………………………1  Essential items for clean delivery & post-partum period was prepared………………2  Place of delivery was identified…….……..3  Emergency funds was arranged……………4  Identified institution with 24 hr EmOC services……………………………………..5  Other specify____________________........6 | |  | |
|  | | | Where did you give your recent childbirth? | | Home……………………………………….1  Health Post………………………………….2  Health Centre……………………………….3  Hospital……………………………………..4  Private Clinic………………………………..5  Other Specify_____________________.......6 | | 37 | |
|  | | | What is your main reason that you did not use health facility? | | Pregnancy was normal……………………1  Short/urgent labour………………………..2  Family did not allow……………………….3  Health facility too far………………………4  No transportation …………………………..5  Successful pervious childbirth without attending health facility …………………….6  Lack of money……………………………….7  Poor road……………………………………..8  Other specify _______________________....9 | |  | |
|  | | | Who assisted your recent childbirth? | | TBA………………………………………..1  Family member……………………………2  Relative…………………………………….3  Health extension worker……………………4  Nurse………………………………………..5  Midwife……………………………………..6  Doctor……………………………………….7  Other specify___________________………8 | | 40 | |
|  | | | Have you had any bad experience in the health facility during your recent childbirth? | | Yes………………………………………..1  No…………………………………………2 | | 40 | |
|  | | | Which of the following you were experienced? ( Tick all appropriate) | | Providers were arrogant, rude and neglectful…………………………………..1  Providers left me alone for long time………2  Providers slapped me during childbirth……3  Adequate privacy was not provided during childbirth……………………………………4  I waited for a long time after reaching the health facility………………………………5  Other specify______________________....6 | |  | |
|  | | | Have you had any history of pregnancy related complications (obstructed labour, Haemorrhage, unsafe abortion, infection and/ Eclampsia)? | | Yes………………………………………...1  No………………………………………….2 | |  | |
| Section V:- Health Facility service utilization | | | | | | | | |
|  | | Have you been charged for the ambulance service? | | Yes…………………………………………1  No…………………………………………..2 | |  | | |
|  | | During your last pregnancy, did you pay any money for ANC in the HF? | | Yes…………………………………………1  No…………………………………………..2 | |  | | |
|  | | During your last pregnancy, did you pay any money for childbirth service in the HF? | | Yes…………………………………………1  No…………………………………………..2 | |  | | |
|  | | Does HEW visit you? | | Yes…………………………………………1  No…………………………………………..2 | |  | | |
|  | | Have you ever been recognised as a model family? | | Yes…………………………………………1  No…………………………………………..2 | |  | | |
| Section VI: Physical accessibility of health facility related questionnaire | | | | | | | | |
|  | How far are the following nearest health facilities from where you live?  HP  HC  District Hospital | | | _______Minutes and ______in kms………..1  _______Minutes and ______in kms………..2  _______Minutes and ______in kms………..3 | |  | | |
|  | What is the available transportation mechanism to take you to the HF? | | | On foot………………………………….1  Motorcycle...……………………………2  Horse/Donkey…………………………..3  Cart ……………………………………...4  Kareza(Amharic)………………………..5  Ambulance ………………………………6  Bajaj/3 wheel car ………………………...7  Other specify___________________.......8 | |  | | |
|  | To your last childbirth what transportation mechanism have you used? | | | On foot...……………………….……….1  Motorcycle …..…………………………2  Horse/Donkey…………………………..3  Cart ……………………………………...4  Kareza(Amharic)………………………..5  Ambulance ………………………………6  Bajaj/3 wheel car ………………………...7  None(delivered at home)…………………8  Other specify___________________........9 | |  | | |
|  | What kind of road do you have from your home to the nearest health facility? | | | Asphalt…………………………………..1  Paved road……………………………….2  Coble stone road…………………………3  Dust road…………………………………4  Other specify……………………………..5 | |  | | |
|  | In your opinion, how do you rate the road to the nearest health facility (name the nearest health facility)? | | | Very bad…..……………………………..1  Bad…..…………………………………...2  Good…..………………………………….3  Very good…..………………………….....4 | |  | | |
